# Supplementary material for: Factors Affecting Nurses’ Health Promotion Behavior during the COVID-19 Pandemic Based on the Information–Motivation–Behavioral Skills Model
Source: Medicina (Kaunas). 2022 May 27;58(6):720. doi: 10.3390/medicina58060720 (PMC9227015; doi:10.3390/medicina58060720)
Supplement: Supplementary file 1 [file medicina-58-00720-s001.zip › medicina-1712307-supplementary.pdf]

Supplement Table S1. Disabilities or Health problems

Variables	Categories	n
Disabilities or Health problems† (n = 118) †	Neck/back pain	77
	Sleep disorders	40
	Emotional problems	15
	Eye/vision	14
	Other	13
	Musculoskeletal diseases	9
	Respiratory diseases	9
	Cardiovascular disease	4
	Arthritis	3
	Hearing disturbance	2
	Cancer	2

† Health problems=Yes 118 participants multiple responses
